# Quorum sensing across bacterial and viral domains

**DOI:** 10.1371/journal.ppat.1009074

**Published:** 2021-01-07

**Authors:** Olivia P. Duddy, Bonnie L. Bassler

**Affiliations:** 1 Department of Molecular Biology, Princeton University, Princeton, United States of America; 2 Howard Hughes Medical Institute, Chevy Chase, United States of America; Tufts Univ School of Medicine, UNITED STATES

## Introduction

Quorum sensing (QS) is a process of cell-to-cell communication that bacteria use to orchestrate collective behaviors in response to changes in cell population density and species composition of the community [1]. QS relies on the production, release, and group-wide detection of and response to extracellular signaling molecules called autoinducers (AIs) [1]. Recent studies demonstrate that bacteria-infecting viruses, called phages, also employ chemical communication to regulate collective activities. Phages can encode exclusive phage QS-like systems, or they can tune into and manipulate their host bacterial QS-mediated communication pathways to optimize the timing of the lysis–lysogeny switch. These research advances suggest that phage-mediated QS signaling and phage eavesdropping on bacterial QS signaling drive bacteria–phage interactions, possibly contributing to mechanisms that shape both phage and bacterial biology [2–6]. Here, we briefly review QS in bacteria, and we summarize recent highlights in chemical communication among phages and across the bacterial and phage domains.

## The bacterial chemical lexicon

**Fig 1:**
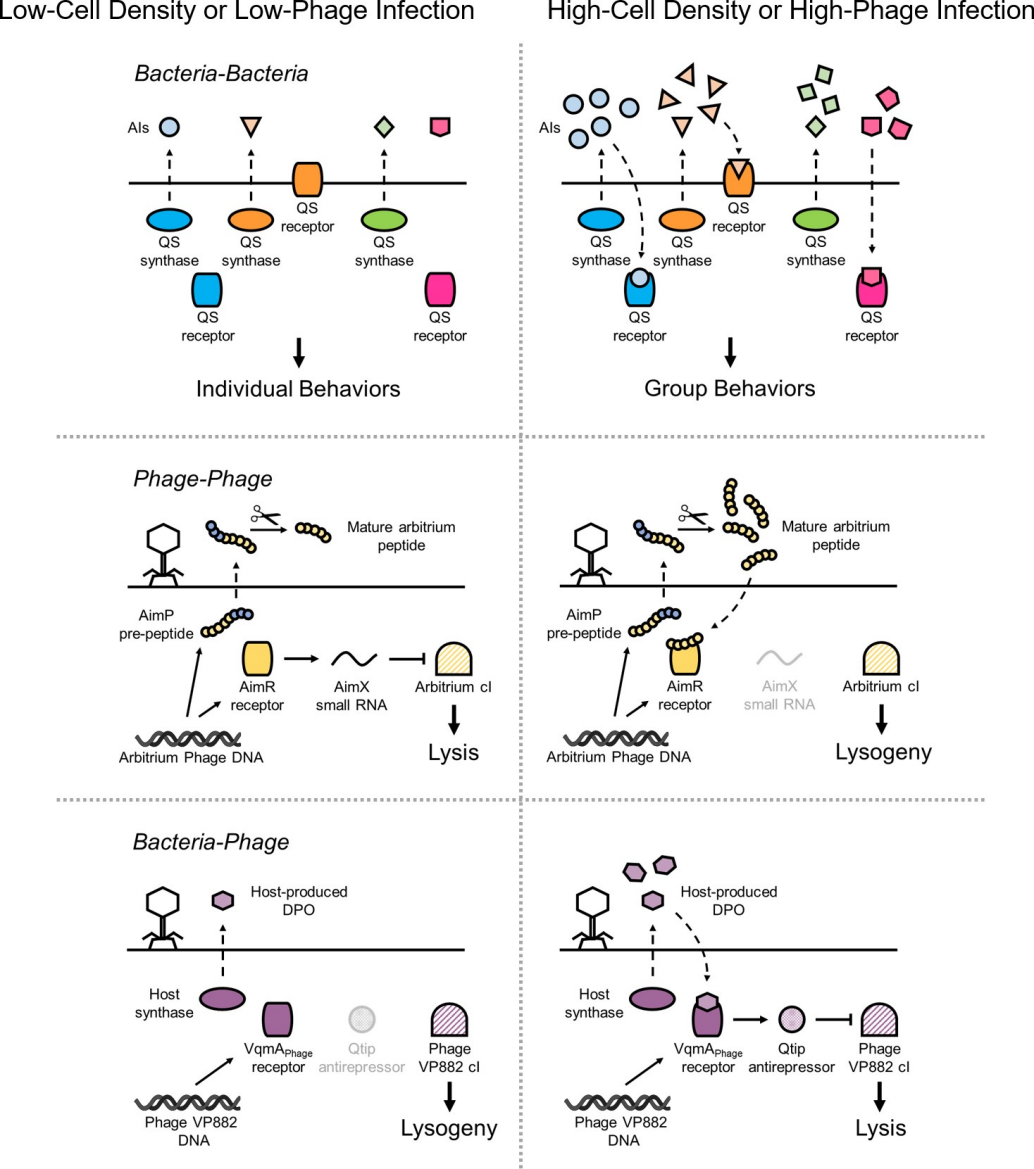
QS-mediated communication. Shown are representative chemical communication systems highlighted in the text that occur between top, bacteria–bacteria; middle, phage–phage; and bottom, bacteria–phage. In each case, the low- and high-cell density or low- and high-phage infection states are shown on the left and right sides, respectively. In each panel, dashed arrows represent release and uptake of AIs, solid arrows represent peptide/protein production or gene regulation, and the horizontal line represents the bacterial membrane. In the middle panel, the scissors signify processing of the signaling peptide. See text for details about each system. AI, autoinducer; QS, quorum sensing.

QS-mediated communication systems are widespread in the bacterial world. QS controls group behaviors including bioluminescence, competence for DNA uptake, virulence factor production, biofilm formation, and the regulation of antiphage defense strategies [1,7,8]. Commonly, bacteria integrate information encoded in multiple AIs, enabling intraspecies, intragenera, and interspecies cell–cell communication (Fig 1, top). Gram-negative bacteria typically use acyl-homoserine lactones (AHLs) as AIs [1]. AHLs are usually produced by LuxI-type synthases and are detected by partner LuxR-type cytoplasmic receptor-transcription factors. Gram-positive bacteria predominantly use oligopeptides as AIs, which are detected by membrane-bound two-component sensor histidine kinases, and the information is relayed to cognate cytoplasmic response regulators [9]. New AIs continue to be discovered expanding our knowledge of the bacterial chemical lexicon. For example, a family of AIs based on rearranged forms of 4,5-dihydroxy-2,3-pentanedione, collectively referred to as autoinducer 2 (AI-2) [10–12], and the pyrazine AI 3,5-dimethyl-pyrazin-2-ol (DPO) [13] are broadly produced among gram-negative and gram-positive bacteria and enable interspecies communication. AI-2 AIs are detected by periplasmic binding proteins homologous to the first known AI-2 receptor, LuxP [12,14–16], and DPO is detected by a cytoplasmic transcription factor called VqmA [13].

Curiously, some bacterial QS systems appear to foster “one-way” conversations (Fig 1, top). In one scenario, bacteria cannot produce an AI but can detect it. For example, neither *Escherichia coli* nor *Salmonella enterica* possess a LuxI-type AI synthase, and therefore, they make no AHL AIs [17]. However, both *E*. *coli* and *S*. *enterica* encode the SdiA LuxR-type receptor that detects exogenously supplied AHLs [17]. Thus, collective behaviors in these bacteria are presumed to be driven by other AHL-producing bacteria in the vicinal community. In a second scenario, bacteria can produce an AI but do not possess an apparent partner AI receptor. This arrangement is relevant to both the AI-2 and DPO AIs. The capacity to produce these AIs is widespread among bacteria; however, few receptors have been shown capable of AI-2 recognition [10,12,15,16], and to our knowledge, among bacteria, only *Vibrio*s possess VqmA DPO-receptors [7]. Thus, presumably, only select bacteria can garner information from these two AI inputs. It remains possible that bacteria make AIs (i.e., AHLs) by atypical routes, and/or they possess unconventional AI-2 and DPO receptors. Alternatively, these asymmetric AI production and detection patterns could confer particular advantages exclusively to subsets of bacteria existing in mixed-species communities.

## Phage lingo

Phages employ two strategies to control their proliferation: dissemination and persistence. Lytic phages, upon entering the bacterial host, replicate and lyse the infected host cells [18]. By contrast, lysogenic or temperate phages can remain dormant in host cells and are passed down via the host cells’ progeny [18]. Importantly, temperate phages can harbor the ability to convert from the lysogenic mode to the lytic mode [18,19]. Seminal studies of phage lambda from *E*. *coli* have guided our understanding of the lysis–lysogeny lifestyle switch [19]. Common to many phages is that inhibition of the phage lytic repressor, called cI, is crucial for launching the phage lytic cascade that drives host cell killing.

Coordination of group behaviors among viruses is far less understood than is the choreography of collective traits in bacteria. Recently, a small-molecule QS-like phage communication process was discovered, termed the “arbitrium system” (Fig 1, middle) [20]. Following phage phi3T infection of *Bacillus* species, a phage-encoded precursor peptide called AimP is produced and secreted. AimP is processed by extracellular proteases into the final arbitrium signaling peptide. The mature peptide is internalized by bacteria, and if they are phage infected, the peptide is detected by the phage AimR receptor, which is a transcription factor. In the unliganded state, AimR binds DNA and activates transcription of the gene encoding the AimX small RNA. AimX represses expression of the arbitrium cI repressor gene, and subsequently, the lytic cascade is deployed [21]. At sufficient concentration, the AimP peptide binds and inactivates AimR. Consequently, *aimX* is not expressed, cI is made and represses lytic development, and lysogeny is established. Thus, newly infecting phages can avoid triggering the lytic cascade when there is low availability of uninfected hosts in the vicinity [20,21].

Arbitrium-like systems exist among numerous phage groups and in conjugative elements, with the majority identified in *Bacillus* species [21]. The native *Bacillus subtilis* conjugation plasmids pLS20 and ICEBs1 use peptide-based signaling systems to regulate expression of plasmid genes [22,23]. Analogous to the phage arbitrium system, accumulation of the plasmid-produced signaling peptide represses conjugation. Thus, DNA transfer is suppressed under conditions when few non-plasmid carrying (i.e., “uninfected”) cells are present.

## A shared bacterial–phage vocabulary

The potential for QS-like chemical communication between bacteria and phages emerged from the discovery that phages can encode homologs of QS components. Specifically, sequencing of the *Clostridrium difficile* temperate phage phiCDHM1 revealed genes homologous to the bacterial accessory gene regulator (Agr) QS system, a peptide-based QS system used by gram-positive bacteria [24]. Phage phiCDHM1 possesses genes encoding predicted homologs of AgrD, AgrB, and AgrC, which are required to produce and secrete the Agr autoinducing peptide [9]. The phage lacks a gene specifying the QS receptor-transcription factor AgrA. The hypothesis is that the phage-produced signal could be detected by the *C*. *difficile* community [24]. Thus, infection of only a few host cells could drive community-wide collective bacterial behaviors. Similarly, DNA sequencing shows that an uncharacterized *Myoviridae* phage encodes a predicted LuxI–LuxR QS pair [25]. While verification is needed, this arrangement could enable two-way interdomain communication: the phage-produced AI could be detected by the bacterial LuxR, and/or the host-produced AI could be detected by the phage LuxR. If so, each entity could control the other’s behavior. Our early knowledge of possible bacteria–phage QS interactions relies primarily on genomic sequencing data. As more viral genomes are sequenced, additional assemblies of phage-encoded QS components are being revealed [25,26]. We anticipate future identification of the outputs controlled by these systems.

A concrete link between host QS and the control of the phage lysis–lysogeny transition is established via the example of Vibriophage VP882 (Fig 1, *bottom*). Specifically, phage VP882 encodes a homolog of the bacterial VqmA DPO-binding QS receptor and transcription factor [27,28]. The phage homolog is called VqmA_Phage_ [28]. When the bacterial-produced DPO AI accumulates at high cell density, VqmA_Phage_ binds DPO. Subsequently, DPO-bound VqmA_Phage_ activates transcription of the phage gene *qtip*, encoding a novel antirepressor, Qtip. Qtip binds and sequesters the phage VP882 repressor, called cI_VP882_, to the cell poles [28,29]. The consequence of Qtip-directed inactivation of cI_VP882_ is derepression of the lytic gene activator Q and expression of genes required for host cell lysis [28]. The notion is that by monitoring the host-produced QS AI, phage VP882 is able to tune the timing of lysis to conditions of high host cell density [28]. Thus, phage VP882 exclusively triggers dissemination from its current host when the probability of infection of the next *Vibrio* cell is maximized [28,30]. Finally, while phage VP882 does not possess the capacity to synthesize DPO, VqmA_Phage_ can activate expression of host-encoded *vqmR*, the transcriptional target of bacterial VqmA [13,28,31]. VqmR is a small RNA that, in *Vibrio cholerae*, regulates genes required for pathogenicity [13,31]. Thus, phage VP882, beyond connecting its own biology to host QS, directly regulates host gene expression, and specifically, host QS-controlled genes.

Observations analogous to those regarding phage VP882 and DPO were recently reported for the *E*. *coli* phage T1 and for *Enterococcus faecalis* temperate phages. Specifically, the administration of synthetic AI-2 to cell cultures induced phage lytic development [32,33]. How the AI-2 input drives phage induction is unknown, and the phage T1 and the *E*. *faecalis* phage genomes harbor no obvious AI-2 receptors. Finally, in *Vibrio anguillarum*, QS represses φH20-like phage p41 lytic development at high cell density, again by an unknown mechanism [34]. We speculate that many more phages can derive information from host-produced QS signals to regulate their lysis–lysogeny transitions.

## Concluding remarks

Here, we have focused on newly discovered QS-mediated chemical interactions between phages and bacteria. These studies reveal that phages, like bacteria, have mechanisms that foster collective processes. From the phage side, using or eavesdropping on QS provides an insidious strategy for phages to optimally prey on bacterial hosts. From the bacterial side, QS-controlled antiphage defense mechanisms provide bacteria enhanced tactics for combatting these very same predators. In particular, at high cell density, QS represses production of cell surface phage receptors [8,35], activates transcription of CRISPR antiphage systems [7,36], and induces phage-inactivating proteases [37,38], all of which defend bacteria against their viral foes. Given that the risk of phage infection escalates with increasing bacterial cell density, placing antiphage defense mechanisms under QS control presumably enables those defenses to be deployed precisely when vulnerability to phage infection is high.

We note that examples also exist of QS-mediated interdomain communication between bacteria and eukaryotes. Specifically, fungi, plant cells, and mammalian cells can synthesize AI mimics that modulate bacterial QS-controlled behaviors [39–42]. Eukaryotic host factors can likewise modulate QS via inactivation or sequestration of bacterial AIs [43–46]. The role of phages in phage–bacterial relationships and in three-way phage–bacterial–eukaryotic partnerships, both harmful and beneficial, represents an exciting research frontier. Given the prevalence of phages in bacterial communities combined with the prevalence of microbiome bacteria in and/or on eukaryotic hosts, defining the contributions of phages to QS could prove central to a comprehensive understanding of the functioning of QS in natural settings.
